# Supplementary material for: Railway fastener defect detection using RFD-DETR: A lightweight real-time transformer-based approach
Source: PLoS One. 2025 Nov 10;20(11):e0331513. doi: 10.1371/journal.pone.0331513 (PMC12599954; doi:10.1371/journal.pone.0331513)
Supplement: Attachment — Submitted filename: Response to Reviewers.docx [file pone.0331513.s001.docx]

**Railway fastener defect detection using RFD-DETR: a lightweight real-time transformer-based approach [PONE-D-25-26834] Response to Reviewers**

Firstly, we are very grateful to the Editor and Reviewers for giving us such a valuable opportunity to revise our manuscript! We appreciate the time and effort that the Editor and the Reviewers for the insightful and constructive feedback. We are convinced that the Reviewers' comments have greatly improved our manuscript and brought us a lot of inspiration.

Based on the Reviewers' comments, we have corrected the errors in the content of the manuscript, and have made extensive alterations to the structure, format, and experimental analyses of our manuscript. In response, we have addressed all of the Reviewers technical concerns with a substantial amount of new data, supported by additional detailed explanations in our revised manuscript.

Below are our point-to-point responses to the Reviewers' valuable comments, including the exact location where the change can be found in the revised manuscript. We use **black bold font** for the Reviewers' comments, normal black font for our responses, black italics for the content in the original manuscript, and *red italics* for the changes in the revised manuscript.

**To Editor**

**1. Please ensure that your manuscript meets PLOS ONE's style requirements, including those for file naming. The PLOS ONE style templates can be found at**

[**https://journals.plos.org/plosone/s/file?id=wjVg/PLOSOne_formatting_sample_main_body.pdf**](https://track.editorialmanager.com/CL0/https:%2F%2Fjournals.plos.org%2Fplosone%2Fs%2Ffile%3Fid=wjVg%2FPLOSOne_formatting_sample_main_body.pdf/1/010f0197633c0744-ad212af5-c694-4093-948f-20a766ceae6a-000000/3cjAnjMWRnd9h5mkRRZG7PxpT9ZlOh4u5lh9riS5WcM=215)  **and**

[**https://journals.plos.org/plosone/s/file?id=ba62/PLOSOne_formatting_sample_title_authors_affiliations.pdf**](https://track.editorialmanager.com/CL0/https:%2F%2Fjournals.plos.org%2Fplosone%2Fs%2Ffile%3Fid=ba62%2FPLOSOne_formatting_sample_title_authors_affiliations.pdf/1/010f0197633c0744-ad212af5-c694-4093-948f-20a766ceae6a-000000/AhbztOLuYit64K_NYrnuH2TFgV25BsXRpJCgri-nsRs=215)

Thank you for your reminder. We have carefully followed the official PLOS ONE LaTeX template **https://journals.plos.org/plosone/s/latex** to prepare our manuscript. The formatting, structure, and file naming fully comply with the journal’s requirements. If there are any further formatting issues, we will be happy to make additional adjustments as needed.

**2. Please note that PLOS ONE has specific guidelines on code sharing for submissions in which author-generated code underpins the findings in the manuscript. In these cases, we expect all author-generated code to be made available without restrictions upon publication of the work. Please review our guidelines at** [**https://journals.plos.org/plosone/s/materials-and-software-sharing#loc-sharing-code**](https://journals.plos.org/plosone/s/materials-and-software-sharing#loc-sharing-code) **and ensure that your code is shared in a way that follows best practice and facilitates reproducibility and reuse.**

Thank you for your reminder. In accordance with PLOS ONE’s code sharing policy, we have made all author-generated code publicly available in a GitHub repository. The repository link and relevant information have been provided in the “Data Availability Statement” and the “Additional Information” section of the submission system. The code is shared without restriction and is intended to facilitate reproducibility and reuse, fully complying with the journal’s guidelines.

**3. Thank you for stating the following financial disclosure:**

**“Huixiang Zhou,**

**National Natural Science Foundation of China(Grant No. 62162028)”**

**Please state what role the funders took in the study.  If the funders had no role, please state: "The funders had no role in study design, data collection and analysis, decision to publish, or preparation of the manuscript."**

**If this statement is not correct you must amend it as needed.**

**Please include this amended Role of Funder statement in your cover letter; we will change the online submission form on your behalf.**

Thank you for your guidance. We have amended the Role of Funder statement as required. Specifically, the funder (Huixiang Zhou) provided financial support for this study and contributed to project supervision. He was not involved in data collection and analysis or preparation of the original draft. The specific contributions of each author are detailed in the Author Contributions section.We have included this amended statement in the Cover Letter for your reference.

**4. We note that you have indicated that there are restrictions to data sharing for this study. PLOS only allows data to be available upon request if there are legal or ethical restrictions on sharing data publicly. For more information on unacceptable data access restrictions, please see** [**http://journals.plos.org/plosone/s/data-availability#loc-unacceptable-data-access-restrictions**](http://journals.plos.org/plosone/s/data-availability#loc-unacceptable-data-access-restrictions)**.**

**Before we proceed with your manuscript, please address the following prompts:**

**a) If there are ethical or legal restrictions on sharing a de-identified data set, please explain them in detail (e.g., data contain potentially identifying or sensitive patient information, data are owned by a third-party organization, etc.) and who has imposed them (e.g., a Research Ethics Committee or Institutional Review Board, etc.). Please also provide contact information for a data access committee, ethics committee, or other institutional body to which data requests may be sent.**

**b) If there are no restrictions, please upload the minimal anonymized data set necessary to replicate your study findings to a stable, public repository and provide us with the relevant URLs, DOIs, or accession numbers. For a list of recommended repositories, please see**

[**https://journals.plos.org/plosone/s/recommended-repositories**](https://journals.plos.org/plosone/s/recommended-repositories)**. You also have the option of uploading the data as Supporting Information files, but we would recommend depositing data directly to a data repository if possible.**

**We will update your Data Availability statement on your behalf to reflect the information you provide.**

Thank you for your attention to data availability. There are no legal or ethical restrictions on sharing the data used in this study. The dataset is publicly available from the Roboflow platform at the following URL:

<https://universe.roboflow.com/sinclair-9xwku/railway-track_fastener_defcts1-gfyj6> The dataset is licensed under CC BY 4.0, and all data necessary to replicate our study findings can be accessed and downloaded from this repository. We have also cited the dataset in the manuscript and provided the relevant information in the Data Availability Statement and Additional Information section of the submission system.

**To Reviewer #1**

**1. The article states that the dataset is sourced from the Roboflow platform at** [**https://universe.roboflow.com/objectdetectiondeeplearning/railwaytrack_fastener_defcts1**](https://universe.roboflow.com/objectdetectiondeeplearning/railwaytrack_fastener_defcts1) **but does not clarify whether all data are publicly available or provide details on access restrictions or contact information. I recommend supplementing a complete data availability statement to specify data access methods or limitations.**

Thank you for your suggestion. In the revised manuscript, we have clarified the data availability statement in the "Dataset Description" section. The dataset used in this study is publicly available from the Roboflow platform at <https://universe.roboflow.com/sinclair-9xwku/railway-track_fastener_defcts1-gfyj6>  and is licensed under CC BY 4.0. We have also cited the dataset as a reference in the manuscript. Please note that the dataset is provided in an augmented form; the original, unaugmented images are no longer available, and the specific augmentation parameters (such as the probability and range of flips, scaling, rotations, etc.) are not documented. Therefore, all training and evaluation in this study were conducted using the provided augmented dataset, and the precise details of the augmentation process cannot be fully reported.

**Location:**

1. *In original manuscript:*

*Section “Experiment and Results“, SubSection "Dataset Description", page 9, lines 351–360.*

*Modified in revised manuscript:*

*Section “Experiment and Results“, SubSection "Dataset Description", page 10, lines 383–395.*

*Section "References" 36, page 22,.*

**2. Experimental results show lower mean average precision for the “missing” and “trackbed-stuff” categories, at 60.46% and 60.03% respectively, possibly due to sample complexity or imbalanced data distribution. The paper does not deeply analyze the model’s robustness under extreme weather, severe occlusion, or complex backgrounds. I suggest adding experiments or discussions targeting these scenarios to validate the model’s practical applicability.**

Thank you for your insightful comment. In the revised manuscript, we have added a new subsection “Verification of Generalizability for Complex Scenarios” in the “Experiment and Results” section. We conducted additional experiments to evaluate the robustness of the model under various extreme weather conditions (fog, rain, snow) and complex backgrounds. The results demonstrate that the improved model maintains strong detection performance and robustness in these challenging scenarios.

**Location:**

1. *Added in revised manuscript:*

*Section “Experiment and Results“, SubSection "Verification of Generalizability for Complex Scenarios", page 10.*

**3.** **The literature review covers YOLO series and Transformer models but includes few references to 2024-2025 studies, such as recent advances in lightweight Transformers or railway defect detection. I recommend incorporating comparisons with the latest research to highlight RFD-DETR’s novelty.**

Thank you for your valuable suggestion. In the revised manuscript, we have updated the Introduction sections to include and discuss several recent studies from 2024 and 2025, focusing on the latest advances in lightweight Transformer models and railway defect detection. In addition, we have incorporated experimental comparisons with these state-of-the-art methods (such as YOLOv10, YOLOv11, YOLOv12, RT-DETRv2, RT-DETRv3, DEIM, RF-DETR, etc.) in both the “Comparisons with Different Detection Models” subsection and the “Visual Analysis” subsection. The visual analysis provides an intuitive comparison of detection results among these models, further highlighting the novelty and advantages of RFD-DETR.

**Location:**

1. *Added in revised manuscript:*

*Section “Introduction“, page 3-4, lines 72–77, lines 90-91, lines 98-111.*

*Section "References" , page 20-22,.*

2. *In original manuscript:*

*Section “Experiment and Results“, SubSection “Comparisons with Different Detection Models”, page 11, lines 404–418.*

*Section “Experiment and Results”, SubSection “Visual Analysis”, page 13, lines 462-487.*

*Modified in revised manuscript:*

*Section “Experiment and Results“, SubSection “Comparisons with Different Detection Models”, page 12-14, lines 477–529, Table 4-5.*

*Section “Experiment and Results”, SubSection “Visual Analysis”, page 18, lines 671-711.*

**4. The article thoroughly describes the technical implementation of the wavelet transform convolution module, cross-scale feature fusion module, and wavelet feature upgrading module but does not adequately explain how these modules specifically enhance feature representation for fastener defect detection. I suggest adding visualization analysis or theoretical derivations to improve model interpretability.**

Thank you for your constructive suggestions. In the revised version, we have added visualization analysis and further theoretical explanations to clarify how the wavelet transform convolution module, cross-scale feature fusion module, and wavelet feature enhancement module improve the feature representation capabilities for fastener defect detection. Specifically, we have added internal ablation experiments for the wavelet convolution module, feature map visualization, and heatmap analysis (e.g., Grad-CAM, Grad-CAM++, XGrad-CAM) to demonstrate the model's improved focus on relevant regions and interpretability in defect detection tasks.

**Location:**

1. *Added in revised manuscript:*

*Section “Experiment and Results“, SubSection “Main Result”, page 15-17.*

**5. The experimental setup describes hardware and hyperparameter settings but omits details on the implementation of data augmentation techniques, such as the proportions or parameters for flips, scaling, and rotations. I recommend including a detailed description of data augmentation in the methods section.**

Thank you for your helpful suggestion. In the revised manuscript, we have clarified the data augmentation process in the “Dataset Description” section. Specifically, we state that the dataset used in this study was directly obtained from the Roboflow platform in an augmented form. The original, unaugmented images are no longer available, and the specific augmentation parameters (such as the probability and range of flips, scaling, rotations, etc.) are not documented in the dataset description. Therefore, all training and evaluation in this study were conducted using the provided augmented dataset, and the precise details of the augmentation process cannot be fully reported.

**Location:**

1. *In original manuscript:*

*Section “Experiment and Results“, SubSection "Dataset Description", page 9, lines 351–360.*

*Modified in revised manuscript:*

*Section “Experiment and Results“, SubSection "Dataset Description", page 10, lines 383–395.*

*Section "References" 36, page 22,.*

**6. The paper proposes optimizing the model through 3D modeling and edge computing but does not specify implementation pathways or potential challenges. I recommend providing more specific future research directions, such as strategies for designing 3D synthetic data generation or model compression techniques for edge devices.**

Thank you for your valuable suggestion. In the revised manuscript, we have expanded the “Conclusion” section to provide more specific future research directions. We now discuss the use of 3D modeling techniques to generate synthetic railway fastener images by constructing realistic 3D models and simulating various defect types, lighting conditions, and backgrounds, which can help create a more diverse and balanced dataset. Additionally, we elaborate on advanced model compression strategies, such as pruning, quantization, and knowledge distillation, to facilitate the deployment of RFD-DETR on edge devices with limited computational resources. We also mention the potential challenges, such as maintaining detection accuracy while reducing model size and ensuring the synthetic data closely matches real-world scenarios.

**Location:**

1. *In original manuscript:*

*Section “Conclusion“, page 13.*

*Modified in revised manuscript:*

*Section “Conclusion“, page 19, lines 728–739.*

**To Reviewer #2**

**1. The authors claim that their method effectively reduces computational complexity and enhances feature extraction capabilities. However, the authors only compared the FLOPS and parameter counts of different models, and the wavelet transform process does not seem to be considered, which is not a very fair comparison.**

Thank you for your insightful comment. In the revised manuscript, we have carefully considered the computational complexity and inference speed introduced by the wavelet transform process, especially the WFU module. In the “Ablation Study” subsection, we present detailed experiments where the WFU module is used independently. The results show that, although the WFU module slightly increases the parameter count, it significantly improves the inference speed (FPS), demonstrating its efficiency in practical deployment. Furthermore, in the “Main Result” subsection, we provide heatmap visualizations to illustrate the advantages of the WFU module in enhancing feature representation and model interpretability. These results confirm that the wavelet transform process not only maintains computational efficiency but also improves detection performance.

**Location:**

1. *Added in revised manuscript:*

*Section “Experiment and Results“, SubSection “Main Result”, page 16-17, lines 590-619.*

1. *In original manuscript:*

*Section “Experiment and Results“, SubSection “Ablation Study”. page 12, lines 446-448.*

*Modified in revised manuscript:*

*Section “Experiment and Results“, SubSection “Ablation Study”, page 17, lines 647–650.*

*Section “Experiment and Results“, SubSection “Ablation Study”, page 17-18, lines 659–666.*

**2. The authors mentioned real-time several times in the title and in the manuscript, however, there is not a single experiment in the manuscript that tests real-time performance.**

Thank you for your valuable comment. In the revised manuscript, we have added the frames per second (FPS) metric to the experimental results to directly evaluate the real-time performance of our proposed model and the comparison models. The FPS results are included in the efficiency comparison tables in the “Comparisons with Different Detection Models” and “Ablation Study” subsections. These results demonstrate that RFD-DETR achieves competitive inference speed while maintaining high detection accuracy, thus validating its real-time capability.

**Location:**

1. *Added in revised manuscript:*

*Section “Experiment and Results“, SubSection “Comparisons with Different Detection Models”, page 13-14, lines 500-529.*

2. *In original manuscript:*

*Section “Experiment and Results“, SubSection “Ablation Study”. page 12, lines 446-448.*

*Modified in revised manuscript:*

*Section “Experiment and Results“, SubSection “Ablation Study”, page 17-18, page 17-18, lines 659–666.*

**3. The author's comparison method is limited to the YOLO series, which is not enough. It is recommended that the author add more than ten latest lightweight comparison methods published in top journals or conferences in the past three years.**

Thank you for your valuable suggestion. In the revised manuscript, we have significantly expanded the scope of our experimental comparisons. In addition to the YOLO series, we have included more than ten recent lightweight object detection methods published in top journals and conferences over the past three years, such as YOLOv10, YOLOv11, YOLOv12, RT-DETRv2, RT-DETRv3, DEIM, RF-DETR, Co-DINO, DINO, LW-DETR, and others. The results of these comparisons are presented in the “Comparisons with Different Detection Models” subsection and the “Visual Analysis” subsection, which further demonstrate the advantages and competitiveness of RFD-DETR.

**Location:**

1. *In original manuscript:*

*Section “Experiment and Results“, SubSection “Comparisons with Different Detection Models”, page 11, lines 405-418.*

*Section “Experiment and Results”, SubSection “Visual Analysis”, page 13, lines 463-487.*

*Modified in revised manuscript:*

*Section “Experiment and Results“, SubSection “Comparisons with Different Detection Models”, page 12-14, lines 477–529.*

*Section “Experiment and Results”, SubSection “Visual Analysis”, page 18-19, lines 671-711.*

**4. Wavelet transform plays a very important role in the manuscript, however, wavelet transform experiments are missing, and the effects of different wavelet basis functions and wavelet transform parameters on the results have not been effectively evaluated.**

Thank you for your constructive suggestion. In the revised manuscript, we have added detailed ablation experiments to evaluate the effects of different wavelet basis functions (such as Haar, db1, db2) and different wavelet decomposition levels on the model’s performance. The results of these experiments are presented in the “Main Result” subsection, where we compare the precision, recall, and mAP metrics under various settings. These additions provide a comprehensive analysis of how different wavelet transform parameters influence detection performance, further validating the effectiveness of the wavelet transform in our method.

**Location:**

1. *Added in revised manuscript:*

*Section “Experiment and Results“, SubSection “Main Result”, page 15, lines 530–554, Table 6-7.*

**5. Some of the technical details (e.g. formula derivation, module structure description) in the paper are lengthy, and the descriptions of the WTConv, CSPPDC and WFU modules take up a lot of space, so the innovations of each module are highlighted in the form of bold or paragraph headings.**

Thank you for your helpful suggestion. In the revised manuscript, we have reorganized the “Methods” section to improve readability and clarity. Specifically, we have used clear paragraph headings for each key module (WTConv, CSPPDC, and WFU) and highlighted the main innovations of each module at the beginning of their respective sections. This structural adjustment makes it easier for readers to quickly grasp the core contributions and technical highlights of our work.

**Location:**

1. *Added in revised manuscript:*

*Section “Methods“, SubSection “Backbone Network Integrated with Wavelet Transform Convolution Module”, page 6, lines 220–223.*

*Section “Methods“, SubSection “Improvement of Cross-Scale Feature Fusion Module”, page 7, lines 270–274.
Section “Methods“, SubSection “Wavelet-transform Feature Upgrade of Neck Module”, page 9, lines 329–334.*

**6. There are inconsistencies in the use of terminology in the paper, e.g. “necking network” should be standardized as “neck module” and “WFU” is interpreted differently in different paragraphs. WFU” is interpreted differently in different paragraphs.**

Thank you for pointing out the inconsistencies in terminology. In the revised manuscript, we have carefully reviewed and standardized all technical terms throughout the paper. Specifically, we have replaced all instances of “necking network” and similar expressions with the unified term “neck module.” Additionally, we have ensured that “WFU” is consistently referred to as the “Wavelet Feature Upgrading (WFU) module” upon first mention, and as “WFU module” thereafter. These changes improve the clarity and consistency of the manuscript. The relevant corrections can be found throughout the manuscript, especially in the “Methods” and “Experiment and Results” sections.

Thank you again for your consideration. We hope that the revised manuscript meets the requirements for publication in PLOS ONE
