# Supplementary material for: Railway fastener defect detection using RFD-DETR: A lightweight real-time transformer-based approach
Source: PLoS One. 2025 Nov 10;20(11):e0331513. doi: 10.1371/journal.pone.0331513 (PMC12599954; doi:10.1371/journal.pone.0331513)
Supplement: Attachment — Submitted filename: Response_to_Reviewers_auresp_2.docx [file pone.0331513.s002.docx]

**Railway fastener defect detection using RFD-DETR: a lightweight real-time transformer-based approach [PONE-D-25-26834]**

**Response to Reviewers**

Dear Editor and Reviewers,

We sincerely thank you for providing us with this valuable opportunity to revise our manuscript. We greatly appreciate the time and effort you have dedicated to reviewing our work and providing insightful and constructive feedback. The reviewers' comments have significantly improved our manuscript and provided us with valuable insights for future research.

Based on the reviewers' comments, we have made comprehensive revisions to address all technical concerns raised. Our revised manuscript includes substantial detailed explanations to support our findings.

Below are our point-by-point responses to each comment, with specific references to the locations of changes in our revised manuscript.

**To Editor - Journal Requirements:**

We have carefully reviewed our reference list to ensure completeness and correctness. After thorough verification, we confirm that:

1. All cited references are current and relevant to our research
2. No retracted papers are included in our reference list
3. All references are properly formatted according to PLOS ONE guidelines

We have adjusted the order of references cited in the main text to ensure they appear in descending chronological order, as recommended by the journal. This improves the manuscript's readability and follows standard academic citation practices.

**To Reviewer #1**

Thank you for your continued valuable feedback and for acknowledging that all major comments have been addressed. We appreciate your recognition of our model's contributions and the improvements made in the revised manuscript. Below are our responses to your additional suggestions:

**1. The article mentions the use of an augmented dataset but lacks detailed descriptions of specific data augmentation methods, such as the proportions or parameters for flipping, scaling, or rotation, which limits the understanding and reproducibility of the data preprocessing process.**

As explained in our Dataset Description section (lines 383-395), the dataset was obtained from Roboflow in an augmented form, and the original augmentation parameters are not documented by the dataset provider. We have transparently reported this limitation in the manuscript.

**2. Despite emphasizing "real-time" performance in the title and text, the original manuscript lacks experiments directly validating real-time performance, such as FPS metrics, with supplementation only in the revised version, indicating incomplete experimental design.**

Thank you for this important observation. You are absolutely correct that real-time performance validation is essential for a model emphasizing real-time capabilities. In the revised manuscript, we have comprehensively addressed this by incorporating Frames Per Second (FPS) metrics throughout our experimental evaluation to directly validate the real-time performance claims.

Specific additions in the revised manuscript:

*Table 5 (Efficiency comparison):* FPS metrics for all compared models, demonstrating that RFD-DETR achieves 47.4 FPS, which meets real-time detection requirements.

*Table 8 (Ablation study):* FPS evaluation for each module combination, showing the impact of different components on inference speed.

*Text analysis:* Discussion of real-time performance trade-offs and validation of the model's suitability for real-time railway fastener detection scenarios.

The FPS results confirm that our proposed RFD-DETR maintains competitive inference speed while achieving superior detection accuracy, thus validating the "real-time" claims in our title and methodology.

**3.** **The initial draft includes limited references to 2024-2025 studies on lightweight Transformers and railway defect detection, failing to fully demonstrate RFD-DETR’s novelty compared to the latest methods. It is recommended to cite: "Optimizing Insulator Defect Detection with Improved DETR Models".**

We appreciate the reviewers' suggestion to cite the latest research findings. In the revised manuscript, we have included the recommended reference “Optimizing Insulator Defect Detection via an Improved DETR Model” in the *Introduction section (lines 114–121)*. This citation serves to demonstrate how the innovative RFD-DETR method compares to and surpasses the latest lightweight transformer-based defect detection methods. The addition of this contemporary reference reinforces the positioning of this manuscript within the current research field and better highlights the novelty of the method we propose.

**4. Although wavelet transform is central to the model, the initial draft lacks systematic experiments on different wavelet basis functions and decomposition levels, limiting a comprehensive evaluation of wavelet transform effects.**

We acknowledge the reviewer's observation that wavelet transform is indeed central to our RFD-DETR model. In the revised manuscript, we have addressed this limitation by adding comprehensive ablation experiments on different wavelet basis functions and various decomposition levels (1-3 levels) in the "*Main Result*" subsection. These systematic experiments provide quantitative evidence of how different wavelet configurations affect the model's performance, offering insights into the optimal wavelet parameters for railway fastener defect detection. This enhancement significantly improves the comprehensiveness of our wavelet transform evaluation and strengthens the experimental foundation of our proposed method.

**5. While WTConv, CSPPDC, and WFU modules are introduced, the article lacks in-depth theoretical analysis or derivation of how these modules specifically enhance feature representation, reducing the interpretability of the model design.**

We appreciate the reviewers' feedback on the theoretical depth of the modules we proposed. In the revised version, we have significantly enhanced the theoretical analysis of the WTConv, CSPPDC, and WFU modules in the “*Main Result*” section. Specifically, we have added comprehensive visualization experiments and theoretical explanations to demonstrate how each module contributes to the enhancement of feature representation. For WTConv, we provide a detailed analysis through ablation comparisons using different wavelet basis functions and different numbers of wavelet decomposition layers, illustrating how the wavelet transform captures multi-scale frequency information. For CSPPDC, we demonstrate its cross-scale feature fusion capabilities through comparative visualization analysis. For WFU, we use heatmaps and Grad-CAM visualizations to detail the mechanism of wavelet feature enhancement. These experimental analyses, combined with theoretical explanations, significantly enhance the interpretability of the model design and provide readers with concrete evidence of how our modules enhance feature representation.

**To Reviewer #2**

We sincerely thank Reviewer #2 for their thorough review and for confirming that all previous comments have been adequately addressed. We appreciate their recommendation for acceptance and are grateful for their constructive feedback throughout the review process.

We believe that the revised manuscript now addresses all the concerns raised by the reviewers and meets the publication standards of PLOS ONE. We have provided comprehensive responses to each comment and made substantial improvements to enhance the manuscript's quality, clarity, and scientific rigor.

We sincerely thank the Editor and Reviewers for their valuable time and constructive feedback, which have significantly improved our manuscript.

Sincerely,

The Authors
